# New Insights on the Diversity, Ecology and Genetic Population Structure of *Anisakis* spp. from Fish and Cetacean Hosts from Northeast Atlantic Waters

**DOI:** 10.3390/ani14233531

**Published:** 2024-12-06

**Authors:** Andrea Ramilo, Helena Rodríguez, Miguel López, Ángel F. González, Alfredo López, Graham J. Pierce, Santiago Pascual, Elvira Abollo

**Affiliations:** 1 Instituto de Investigaciones Marinas, Consejo Superior de Investigaciones Científicas, IIM-CSIC, 36208 Vigo, Spain; 2 Departamento Biologia, ESAM, Universidad de Aveiro, Campus Universitario de Santiago, 3810-193 Aveiro, Portugal; 3 Coordinadora para o Estudo dos Mamíferos Mariños (CEMMA), 36380 Nigrán, Spain

**Keywords:** *Anisakis* spp., cetacean, fish, genetic structure, FAO27

## Abstract

In the last 25 years, nematode parasites of the genus *Anisakis* have attracted international attention from various socio-economic sectors, with serious concern about the impact of these parasites on seafood quality and safety, and public health (related to both zoonoses and allergy). A knowledge-based understanding of the population structure of *Anisakis* spp. is useful to provide valuable data about the infection dynamics, host specificity and its ability to adapt to local environments and to climate change by adapting to the food-web structure. This study first aimed to investigate the taxonomic biodiversity of *Anisakis* spp. collected from commercial fish and cetacean species from the most significant Northeast Atlantic fishing grounds and evaluate the ecological connections of *A. simplex* and *A. pegreffii* (L3 and adults) in cetaceans and fish from NW Spain, through the analysis of their genetic diversity and population structure. A total of 1399 *Anisakis* spp. L3 larvae from six fish species and 475 adults from six cetacean species were identified using the ITS rDNA region as a molecular marker. Molecular identification allowed for the first detection of *A. berlandi* in European waters parasitizing the long-finned pilot whales *Globicephala melas* and the first report of *A. nascettii* and *A. zhiphidarum* infecting the striped dolphin *Stenella coeruleoalba,* as well as the identification of *A. simplex, A. pegreffii* and the hybrid genotype between both species. The analysis of the mitochondrial cytochrome oxidase 2 gene of *A. simplex* and *A. pegreffii*, the most prevalent species in FAO area 27, revealed panmictic populations for both species with high haplotype diversity. The predatory–prey relationship involving two major fish species (European hake and blue whiting) and the common dolphin appears to provide an important mechanism for maintaining genetic diversity and structure in major *Anisakis* species in the NE Atlantic.

## Simple Summary

Nematode parasites of the genus *Anisakis* are among the most prevalent parasites of fish exploited by major European fisheries, and they have an important impact on seafood quality and safety, causing significant economic losses and constituting an important public health risk for consumers. The aim of this study is to update the taxonomic biodiversity of *Anisakis* spp. collected from important commercial fish species and several species of cetaceans from the most significant Northeast Atlantic fishing grounds, as well as to provide new information about the population genetic diversity of the main species in FAO27, *A. simplex* and *A. pegreffii*. These data enhance the knowledge of the ecological connections of these parasites between their intermediate/paratenic hosts (fish) and definitive hosts (cetaceans).

## 1. Introduction

Nematodes of the genus *Anisakis* Dujardin, 1845 (Rhabditida, Anisakidae) are among the most prevalent parasites of fish exploited by major European fisheries. These worms also constitute an important public health risk for consumers [1], since they result in both zoonotic and allergic concerns [2,3,4,5]. The life cycle is indirect, involving several hosts at different trophic levels of marine food webs worldwide. The adult stage of *Anisakis* spp. parasitizes the digestive tracts of marine mammals, mainly toothed and baleen whales. Embryonated eggs are released with cetacean faeces into the water column, where the first- (L1) and second-stage larva (L2) develop inside the egg. L2 is ingested by crustaceans (euphausiids and amphipods) or small zooplanktivorous fish and moults to the third stage (L3) until the host is eaten by a fish or cephalopod (intermediate/paratenic hosts). Cetaceans feed on parasitized fish or cephalopods and, in their stomach, the L3 larvae moult into fourth-stage larvae (L4) and develop into sexually mature adults [6,7,8].

Genetic studies of *Anisakis* spp. have contributed to clarifying the taxonomy of morphologically indistinguishable L3 larvae [9,10,11,12]. Recent phylogenetic studies have proposed that the genus *Anisakis sensu stricto* should include only five of the nine species identified up to that moment, although these changes have not yet been completely accepted: *Anisakis simplex sensu stricto*, *A. pegreffii*, *A. berlandi*, *A. ziphidarum* and *A. nascettii*. The species *A. brevispiculata*, *A. paggiae* and *A. physeteris* were assigned to the genus *Skrjabinisakis*, whereas *A. typica* was reassigned to the genus *Peritrachelius* [13,14].

In the Northeast Atlantic (FAO area 27), several drivers are involved in determining the geographic range of *Anisakis* species [7], playing a crucial role in the distribution patterns of their definitive hosts (cetaceans) and intermediate/paratenic hosts (fish, squid and planktonic crustaceans) as well as the trophic links established among them. Numerous epidemiological studies of *Anisakis* spp. larvae in their intermediate/paratenic hosts in the Northeast Atlantic waters have been published (e.g., [6,7,15,16,17,18,19,20]), but studies of *Anisakis* spp. in their adult hosts remain scarce [8,21,22]. *A. simplex* is the most prevalent species, exhibiting high densities in numerous paratenic and definitive hosts [6,8,9,15,18,21,23,24]. In fish from the Atlantic coast of Spain and Portugal, *A. simplex* coexists with *A. pegreffii*, and hybrids between *A. simplex* and *A. pegreffii* have also been found in areas where the two species are sympatric [9,25,26]. Several molecular markers have been described to carry out the differentiation between both *Anisakis* species and their hybrids, including, among them, PCR-RFLPs profiles of the ITS rDNA region and nuclear and mitochondrial marker sequencing or the analysis of panels of microsatellite loci. The ITS1 marker is considered by some authors to be more sensible than other molecular markers to detect F1 hybrids and later-generation backcrosses [9,17,27,28]. Beyond the taxonomic classification, studies of the genetic population structure of parasites, including *Anisakis* spp., can provide valuable data about the dynamics of infection, their host specificity and their ability to adapt to local environments and to climate change [29,30,31]. Furthermore, since the life cycle of *Anisakis* spp. is highly dependent on the food-web structure [7], new evidence on the genetic networks between L3 larvae and adults of *Anisakis* parasitizing intermediate and definitive hosts could also provide valuable information about the population genetic structure and ecology of their hosts, and they could even be used as biological tags for stock identification of fish and cetacean species [32,33,34].

The aims of this work were to (1) update the taxonomic biodiversity of *Anisakis* spp. specimens collected from six important commercial fish species from the most significant Northeast Atlantic fishing grounds and specimens recovered from cetaceans stranded along the Galician coast (NW of Spain) and (2) establish the population genetic diversity and structure of *A. simplex* and *A. pegrefffii*, focusing on the ecological connections of these parasites from different fish and cetacean hosts.

## 2. Materials and Methods

### 2.1. Sample Collection

**Figure 1:**
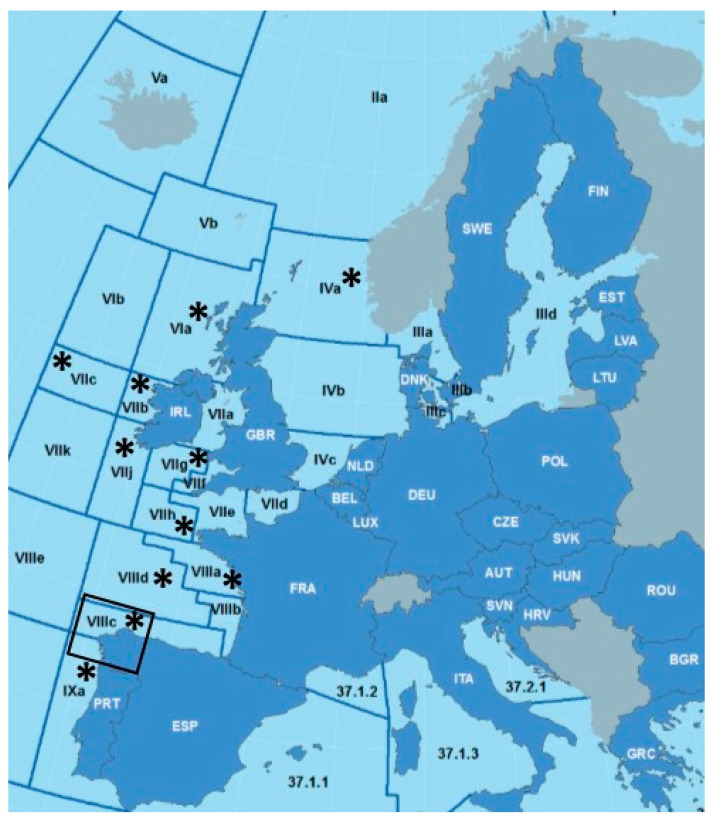
Sampling areas (*) according to the ICES (International Council for the Exploration of the Sea) division areas: IVa (northern North Sea), VIa (northwest coast of Scotland and North Ireland), VIIb (west of Ireland), VIIc (Porcupine Bank), VIIg (Celtic Sea/north), VIIh (Celtic Sea/south), VIIj (southwest of Ireland/east), VIIIa (Bay of Biscay/north), VIIIc (Bay of Biscay/south), VIIId (Bay of Biscay/offshore) and IXa (Portuguese Waters/east). The Galician coast is marked by a square.

A total of 475 adult specimens of *Anisakis* spp. were collected from 51 cetaceans stranded on the Galician coast (NW of Spain) (Figure 1, Table 1) between 2008 and 2022 by the NGO “Coordinadora para o Estudio dos Mamíferos Mariños” (CEMMA). In this sampling, 30 common dolphins, *Delphinus delphis* (DDE), 8 striped dolphins, *Stenella coeruleoalba* (SCO), 7 harbour porpoises, *Phocoena phocoena* (PPH), 3 bottlenose dolphins, *Tursiops truncatus* (TTR), 2 long-finned pilot whales, *Globicephala melas* (GME) and 1 Risso’s dolphin, *Grampus griseus* (GGR), were necropsied (Table S1). A subsample of adult nematodes parasitizing each cetacean was collected, and the parasites were washed in phosphate-buffered saline (Thermo Scientific, Waltham, MA, USA) and then stored frozen at −20 °C for subsequent molecular analysis. Additionally, 1399 *Anisakis* spp. third-stage larvae collected between 2018 and 2020 from different fish species (*Merluccius merluccius*, *Lepidorhombus* spp., *Lophius budegassa*, *Lophius piscatorius*, *Micromessistius poutassou* and *Engraulis encrasicolus*) from Northeast Atlantic fishing grounds were included in this study (Figure 1, Table 2). All *Anisakis* larvae were donated by the Technical Unit of the Marine Biobank at the Institute of Marine Science (UTB-IIM-CSIC, Vigo, Spain).

### 2.2. Genetic Identification of Anisakis spp.

Genomic DNA purification of each nematode was performed using a custom version of the Maxwell HT Purefood kit (Promega) on TECAN’s robotics platform. The entire ITS region (ITS-1, 5.8S rDNA gene and ITS-2) was amplified using the primers NC5 (5′- GTAGGTGAACCTGCGGAAGGATCATT-3′) and NC2 (5′-TTAGTTTCTTTTCCTCCGCT-3′) [35]. PCR mixtures were also performed in the robotic workstation in a total volume of 25 μL containing PCR buffer at 1× concentration, 0.2 mM nucleotides (Thermo Scientific, Waltham, MA, USA), 0.3 μM of each primer, 0.025 U μL^−1^ Dream Taq DNA polymerase (Thermo Scientific) and 1 μL of genomic DNA (50–100 ng). A negative control was included in all PCR amplifications.

PCR products were separated on a 2% agarose gel stained with 5 μL/100 mL RedSafeTM nucleic acid staining solution and scanned in a GelDoc XR documentation system (Bio-Rad Laboratories, Hercules, CA, USA). PCR products were cleaned for sequencing using ExoSap-IT (Thermo Fisher Scientific, Waltham, MA, USA), as supplied by the manufacturer, and the sequencing was performed using the company STABVIDA (Caparica, Portugal). Sequences were edited using ChromasPro v.2.1.10 (Technelysium Pty Ltd., South Brisbane, Australia). Two diagnostic nucleotide sites of the ITS1 region, positions 278 and 294, were identified in order to differentiate *A. simplex* (T nucleotide in both sites), *A. pegreffii* (C in both of them) and heterozygotes (two overlapping C/T peaks in both positions) [9]. All generated sequences were also assessed for similarity against known sequences using BLAST (Basic Local Alignment Search Tool) from the National Center for Biotechnology Information (NCBI, Bethesda, MD, USA).

### 2.3. Genetic Diversity and Haplotype Analysis

The genetic diversity analysis was performed with the identified species *A. simplex* and *A*. *pegreffii*. Genomic DNA was amplified at the mitochondrial cytochrome oxidase 2 gene (mtDNA *cox2*), using a 211F/210R pair of primers described by Nadler and Hudspeth [36]. PCR reactions were performed as described above and under the following reaction parameters: 95 °C for 5 min, 35 cycles at a melting temperature of 95 °C for 30 s, an annealing temperature of 48 °C for 45 s and an extension temperature of 72 °C for 1 min, followed by a final extension period of 72 °C for 7 min. PCR products were also sequenced and analysed as described above.

Multiple alignments of the sequences achieved were constructed using MEGA 7 [37] and analysed in the software DnaSP v6 [38] in order to know the genetic diversity of *A.* simplex (s.s.) and *A. pegreffii* populations in the sampling area. Three sets of mtDNA *cox2* sequences for each species were defined in DnaSP v6: (1) *A. simplex* or *A. pegreffii* adult sequences grouped by cetacean species; (2) *A. simplex* or *A. pegreffii* L3 larvae sequences from different fish species; and (3) *A. simplex* or *A. pegreffii* sequences from IXa and VIIIc divisions from both cetacean and fish species. The number of haplotypes (Nh), the haplotype diversity (H_d_), nucleotide diversity (π), the number of segregating sites (S) and the average number of nucleotide differences (K) were calculated by DnaSP v6 for all defined sets. Median-joining haplotype networks [39] were constructed using PopART (http://popart.otago.ac.nz, accessed on 20 May 2024). A null hypothesis of population panmixia was tested using two neutrality tests, Tajima’s D [40] and Fu’s Fs [41], performed in Arlequin v3.5.2. software [40] with 1000 simulations to analyse the randomness of the DNA sequence evolution by the verification of the null hypothesis of selective neutrality (expected with population expansion). Positive Tajima D and Fu Fs values suggest a low genetic diversity, which means a recent population bottleneck, whereas negative values signify high genetic diversity, as would be expected from a recent population expansion. In addition, the genetic structure of the *A. simplex* and *A. pegreffii* populations was also evaluated in Arlequin v3.5.2. software [42] by a hierarchical analysis of molecular variance (AMOVA) and by pairwise comparisons of F_st_ [43] values between populations, calculated with 1000 permutations. F_st_ values range from F_st_ = 1, indicating complete differentiation among populations, to F_st_ = 0, which indicates no differentiation among populations.

## 3. Results

### 3.1. Taxonomic Identification of Anisakis spp. from Cetaceans

**Figure 2:**
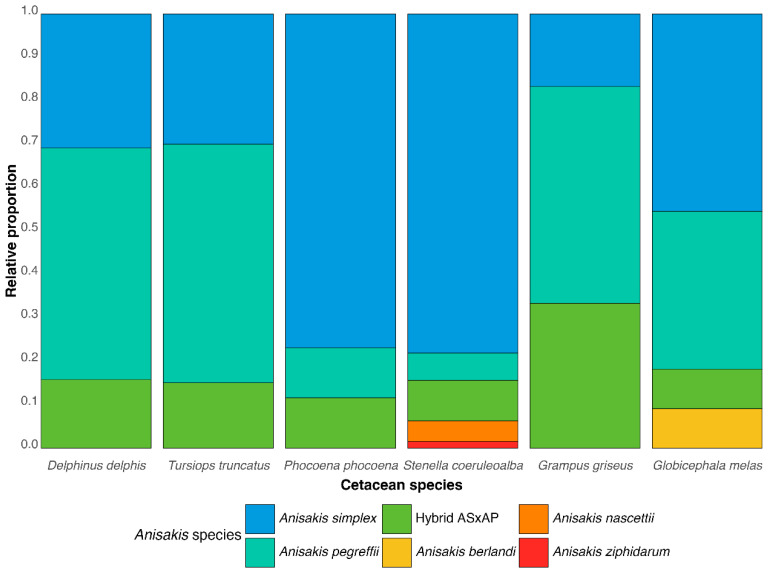
Relative proportions of *Anisakis* species infecting cetacean populations stranded in NW Spain.

Amplification of the entire ITS and 5.8S regions, rendering a PCR product of about 905 bp, was positive for 399 individuals out of 475 adult nematodes analysed. Blast analysis showed that ITS sequences shared 99.5–100% nucleotide identity with sequences of five anisakid species and a hybrid genotype previously deposited in the GenBank. It is noteworthy that most of the stomachs analysed (86.1%) showed sympatric infections by up to three *Anisakis* species. In the common dolphin, the most abundant species was *A. pegreffii* (53.3%), followed by *A. simplex* (30.9%) and the hybrid genotype between both species (15.8%) (Table 1; Figure 2). Co-infection by *A. simplex* and *A. pegreffii* was detected in 19 of the 30 stomachs analysed (63.3%). Infections by *A. simplex, A. pegreffii* and the hybrid genotype were also recorded in nine stomachs (34.6%). Noticeably, striped dolphins were parasitized by a greater number of anisakid species. *A. simplex* was the most abundant (78.1%), followed by the hybrid genotype between *A. simplex* and *A. pegreffii* (9.4%), *A. pegreffii* (6.3%), *A. nascettii* (4.7%) and *A. ziphidarum* (1.6%). Of the eight striped dolphins examined, two showed co-infection by *A. simplex*, *A. pegreffii* and the hybrid genotype, and one was co-infected by *A. simplex*, *A. nascettii* and *A. ziphidarum.* Nematodes collected from the stomachs of the harbour porpoise were identified as *A. simplex* (76.92%), *A. pegreffii* (11.5%) and the hybrid genotype between *A. simplex* and *A. pegreffii* (11.5%), also recording three stomachs parasitized by both species. Three *Anisakis* species, *A. simplex* (45.5%), *A. pegreffii* (36.4%) and *A. berlandi* (9.1%), and the hybrid genotype (9.1%) were identified as infecting the same stomach of a long-finned pilot whale. *Anisakis simplex* (30%)*, A. pegreffii* (55%) and the hybrid genotype (15%) were also identified as parasitizing bottlenose dolphins and were recorded in syntopy in two stomachs. The stomach of Risso’s dolphin was parasitized by *A. simplex* (16.7%), *A. pegreffii* (50.0%) and the hybrid genotype (33.3%).

### 3.2. Taxonomic Identification of Anisakis spp. from Fish Species

A total of 1262 specimens were identified as *A. simplex* (90.2%), 81 as *A. pegreffii* (5.8%) and 56 as the hybrid genotype (4.0%) (Table 2). *A. simplex* was the predominant species in all fish species from all ICES divisions sampled (Figure 1).

All *Anisakis* larvae recovered from *Lepidorhombus* spp. from VIa, VIIb, VIIg and VIIj were identified as *A. simplex*. Similar results were obtained for *Lophius budegassa* and *L. piscatorius* for these divisions, in which *A. simplex* was the predominant species, although, in Via, the hybrid genotype was also identified (2.1% and 9.1%, respectively). Identification of parasites from *M. poutassou* from sympatric areas IXa and VIIIc showed that the predominant species was *A. simplex* (62.5% and 82.9%, respectively), but *A. pegreffii* represented 29.2% in IXa and 13.6% in VIIIc. The hybrid genotype was also identified (8.3% in IXa and 3.6% in VIIIc). In the case of *E. encrasicolus* from VIIIc, all *Anisakis* larvae analysed were identified as *A. simplex*, although only five specimens were sampled. In *M. merluccius*, the results showed that 88.3% of the larvae were *A. simplex*, 6.3% were *A. pegreffii* and 5.4% belonged to the hybrid genotype. In divisions IXa, VIIIa and VIIIc, co-infection by *A. simplex* (59.8%, 88.7% and 73.2%, respectively), *A*. *pegreffii* (25.4%, 4.2% and 16.5%, respectively) and hybrids (14.8, 7.0% and 10.2%, respectively) was found. In the remaining divisions, the percentage values for *A. simplex* ranged between 96.0 and 98.7, and hybrids made up between 1.3% and 4.1%. *A. pegreffii* was not recorded in these ICES areas.

### 3.3. Genetic Diversity and Population Structure of A. simplex

A total of 936 mtDNA *cox2* sequences of *A. simplex* (L3), obtained from the six fish species studied, were used for genetic diversity analysis. The alignment of all *A. simplex* sequences (483 sites) contained 145 variable sites (S) and resulted in 350 haplotypes (Table 3). The overall value of haplotype diversity (Hd) was 0.940, the value of nucleotide diversity (π) was 0.00686, and the value of the average number of nucleotide differences (K) was 3.31167. The genetic indices calculated for *A. simplex* from each fish species showed a similar haplotype diversity for all of them, ranging between 0.931 for *Lepidorhombus* spp. and 0.946 for *L. piscatorius*, except for those of *E. encrasicolus*, which showed lower Hd values (0.833), although only four specimens were analysed. Similar π values were also obtained for all fish species, ranging between 0.00621 for *E. encrasicolus* and *L. piscatorius* and 0.00708 for *M. poutassou*. Neutrality test statistics, Tajima’s D and Fu’s Fs, both showed statistically significant negative values (*p* < 0.05 and *p* < 0.02, respectively) for all fish species, except for E. encrasicolus; thus, the null hypothesis of a constant population size (i.e., the population evolves according to the infinite-site model and all mutations are selectively neutral) was rejected (Table 3).

**Figure 3:**
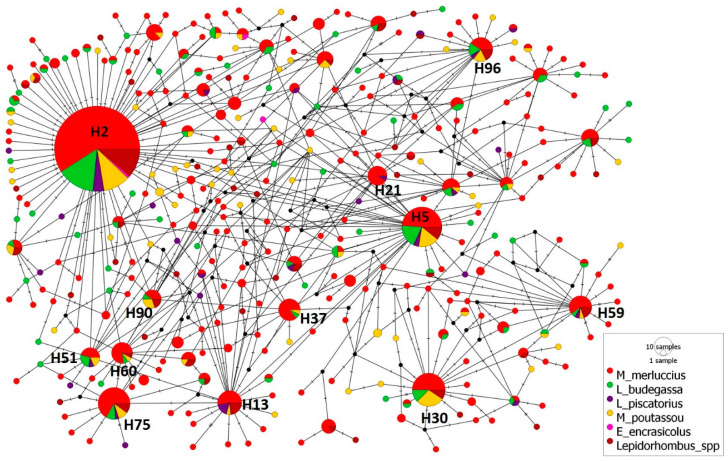
Median-joining haplotype network of *A. simplex* mtDNA *cox2* sequences obtained from 6 fish species. The circles’ size represents the frequency of each haplotype. Hatch marks show the number of mutations distinguishing the haplotypes. The major haplotypes (including more than 10 sequences) are reported. Black points indicate missing haplotypes.

The median-joining haplotype network of the 936 mtDNA *cox2* sequences of *A. simplex* from six fish species studied (Figure 3) shows the 350 haplotypes obtained by DNAsp. Their structure showed that the haplotype H2 was clearly the most common, being represented by 215 sequences (22.97% of the total), and it was the only haplotype shared by *A. simplex* from the six fish species. The relative frequency of occurrence for this haplotype was similar for the parasites from *M. merluccius*, *L. budegassa*, *L. piscatorius* and *Lepidorhombus* spp., ranging between 0.230 and 0.253, but was lower for those from *M. poutassou* (0.193) and higher for *A. simplex* from *E. encrasicolus* (0.5). Other haplotypes represented by more than 1% of the total number of sequences were H5, H59, H75 and H96, shared between *A. simplex* from all fish species except *E. encrasicolus*, and H30, H21, H37, H13, H90, H60 and H51, shared only between those from some species. Moreover, the median-joining haplotype network showed a high number of unique haplotypes (294 haplotypes, 84% of the total) for some A. simplex of the six fish species analysed and, in all case cases, represented by only one or two sequences. Thus, A. simplex from M. merluccius was the species with the highest number of unique haplotypes (77.82% of the total haplotypes for this species), followed by those from M. poutassou (61.19%). For the other fish species, the percentage of unique haplotypes ranged between 33.33% for A. simplex from E. encrasicolus and 49.02% for the parasites from Lepidorhombus spp.

The population structure of *A. simplex* from *M. merluccius*, *Lepidorhombus* spp., *L. budegassa*, *L. piscatorius*, *M. poutassou* and *E. encrasicolus* was analysed with AMOVA, showing that 100% of genetic variation was explained by differences within populations. Pairwise genetic differentiation among *A. simplex* sequences of these species was very low: Fst values ranged between 0 and 0.00109, and the differences between fish species were not statistically significant.

A total of 104 mtDNA *cox2* sequences was obtained from *A. simplex* recovered from cetaceans stranded along the Galician coast (divisions IXa and VIIIc, Figure 1). The alignment of all sequences (473 sites) resulted in 60 haplotypes, containing 46 variable sites (S) (Table 4). The overall value of haplotype diversity (Hd) was 0.935, that of nucleotide diversity (π) was 0.00588, and the average number of nucleotide differences (K) was 2.78099. The genetic indices calculated for *A. simplex* from each cetacean species all showed a similar haplotype diversity, ranging between 0.915 for DDE and 1.000 for TTR, with π values ranging between 0.00000 for GME and 0.00810 for TTR. Neutrality test statistics, Tajima’s D and Fu’s Fs, were calculated for sequences from A. simplex from DDE, SCO and PPH only, since TTR and GME were represented by a low number of sequences. In three species, Tajima’s D and Fu’s Fs showed negative values, and the differences were statistically significant (*p* < 0.05 and *p* < 0.02, respectively), the exception being PPH for which the Tajima D value was not significant (*p* = 0.083). Overall, the null hypothesis is rejected, i.e., the population evolves according to the infinite-site model and all mutations are selectively neutral.

The population structure of *A. simplex* from DDE, PPH and SCO was analysed with AMOVA, showing that 99.71% of genetic variation was explained by differences within populations, and 0.29% by differences among populations. Pairwise genetic differences between *A. simplex* sequences from these cetacean species were not statistically significant, with Fst values ranging between −0.01054 and 0.01194.

**Figure 4:**
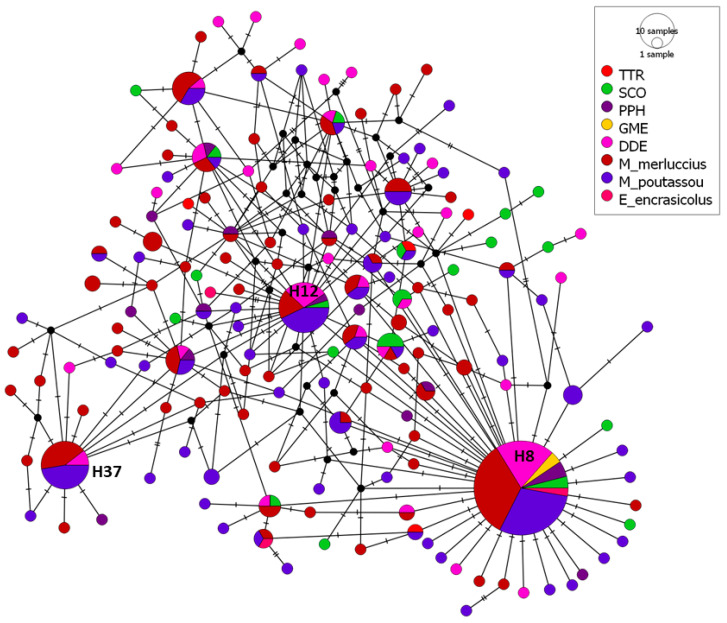
Median-joining haplotype network of *A. simplex* mtDNA *cox2* sequences obtained from cetacean and fish species from divisions VIIIc and IXa. The circles’ size represents the frequency of each haplotype. Hatch marks show the number of mutations distinguishing the haplotypes. Haplotypes shared between species are reported. Black points indicate missing haplotypes. TTR: *Tursiops truncatus*; SCO: *Stenella coeruleoalba*; PPH: *Phocoena phocoena*; GME: *Globicephala melas*; and DDE: *Delphinus delphis*.

Finally, the 104 mtDNA *cox2* sequences of *A. simplex* from five cetacean species were represented by a median-joining haplotype network, together with 243 mtDNA *cox2* sequences of *A. simplex* from *M. merluccius* (125 sequences), *M. poutassou* (114) and *E. encrasicolus* (4) from IXa and VIIIc divisions (Figure 4). A total of 162 haplotypes were obtained, with 136 of them (83.95%) being unique haplotypes for some of the species studied. These unique haplotypes represented 33.33% of total haplotypes from *E. encrasicolus*, 35.29% of total haplotypes from PPH, 50% of total haplotypes from TTR and between 60% and 69% for the other species. No unique haplotype was found for GME, although only three sequences were included. The network showed one haplotype, H8, to be clearly predominant and it was the only haplotype shared by all species (except for TTR). H8 was represented by 74 sequences (21.34% of the total sequences) with relative frequencies of 1 for GME, 0.5 for *E. encrasicolus* and between 0.115 and 0.278 for the other species. The other more common haplotypes (represented by more than 10 sequences) were common only among some of the species: H12 (21 sequences, 6.05% of total sequences) and H37 (19 sequences, 5.48%).

### 3.4. Genetic Diversity and Population Structure of A. pegreffii

A total of 60 mtDNA *cox2* sequences from A. *pegreffii* (L3) were obtained from *Merluccius merluccius* (38) and *Micromesistius poutassou* (22) from VIIIa, VIIIc and IXa divisions. DNAsp results showed that the alignment of all *A. pegreffii* sequences (483 sites) contained 34 variable sites (S) and resulted in 22 haplotypes (Table 5). For this species, Hd values were lower than those obtained for *A. simplex*, for both *M. merluccius* (0.886) and *M. poutassou* (0.831), whereas π and K values were higher for *A. pegreffii* than for *A. simplex* in *M. merluccius* (0.00915 and 4.41963, respectively). Neutrality test statistics, Tajima’s D and Fu’s Fs, showed negative values for both species, which were not statistically significant except in the case of Tajima’s D value for M. merluccius. (Table 5).

**Figure 5:**
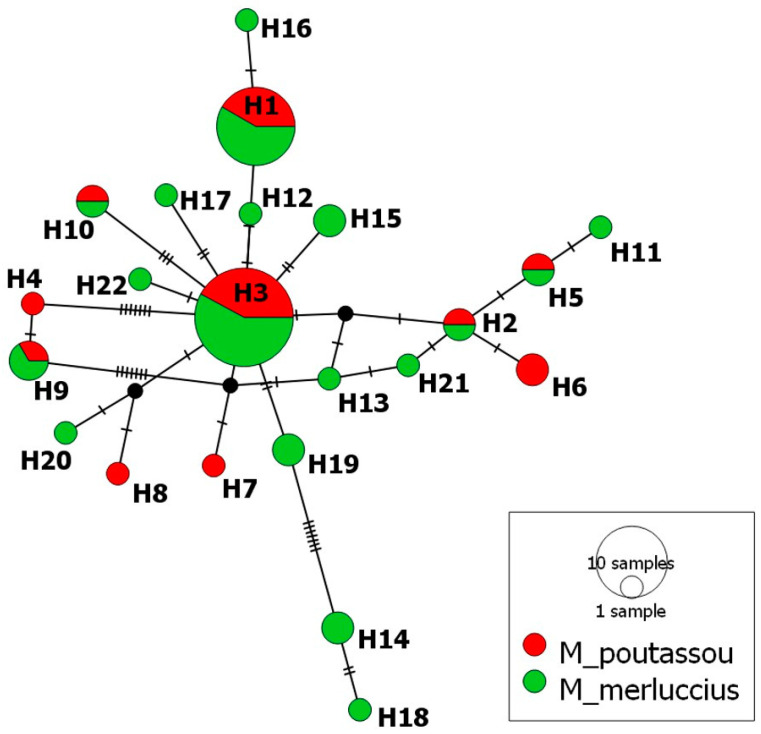
Median-joining haplotype network of *A. pegreffii* (L3) mtDNA *cox2* sequences obtained for *M. poutassou* and *M. merluccius*. Circles’ size represents the frequency of each haplotype. Hatch marks show the number of mutations distinguishing the haplotypes. Black points indicate missing haplotypes.

The median-joining haplotype network of mtDNA *cox2* haplotypes of *A*. *pegreffii* (Figure 5) showed that 6 of 22 haplotypes were shared between the parasites from *M. merluccius* and those from *M. poutassou* (27.27% of total haplotypes). Two of them, H3 and H1, were clearly the most common, represented by 19 and 12 sequences, respectively, with relative frequencies of 0.227 for *A. pegreffii* collected from *M. poutassou* and 0.184 for those from *M. merluccius* in the case of H1, and 0.364 for those from *M. poutassou* and 0.289 for *A. pegreffii* from *M. merluccius* in the case of H3. Sixteen *A. pegreffii* haplotypes were unique to one of the two species studied (72.72% of total haplotypes), representing 66.66% of haplotypes found in *M. merluccius* and 40% of haplotypes of *M. poutassou*.

The population structure of *A. pegreffii* from *M. merluccius* and *M. poutassou*, analysed with AMOVA, was similar to that for *A. simplex*. Thus, 100% of genetic variation was explained by differences within populations. The pairwise genetic difference between these two fish species, for *A. pegreffii* sequences, was not statistically significant (Fst was zero).

A total of 139 mtDNA *cox2* sequences was obtained from *A. pegreffii* (adults) recovered from cetaceans stranded along the Galician coast. These sequences were aligned (490 sites) and analysed by DNAsp, resulting in 42 haplotypes, and containing 47 variable sites (S) (Table 6). The overall value of haplotype diversity (Hd) was 0.896, that of nucleotide diversity (π) was 0.00979, and the average number of nucleotide differences (K) was 4.79575. The genetic indices calculated for *A. pegreffii* from each cetacean species showed the maximum values of Hd (1) for SCO, PPH, GME and GGR, although only 2–3 sequences from each of these species were included in this analysis; Hd was lower for TTR (0.964) and for DDE (0.879). π values were higher for TTR, PPH and GGR (ranging between 0.01039 and 0.02449) and lower than 0.01 for the remaining species. Neutrality test statistics, Tajima’s D and Fu’s Fs, for sequences of A. pegreffii from DDE and TTR showed negative values and were not statistically significant (Table 6).

The population structure of *A. pegreffii* from the six cetacean species was analysed with AMOVA, showing that 97.69% of genetic variation was explained by differences within populations and 2.31% of genetic variance by differences among populations. Pairwise genetic differences between cetacean species for *A. pegreffii* sequences were not statistically significant (Fst values ranging between 0 and 0.09344).

**Figure 6:**
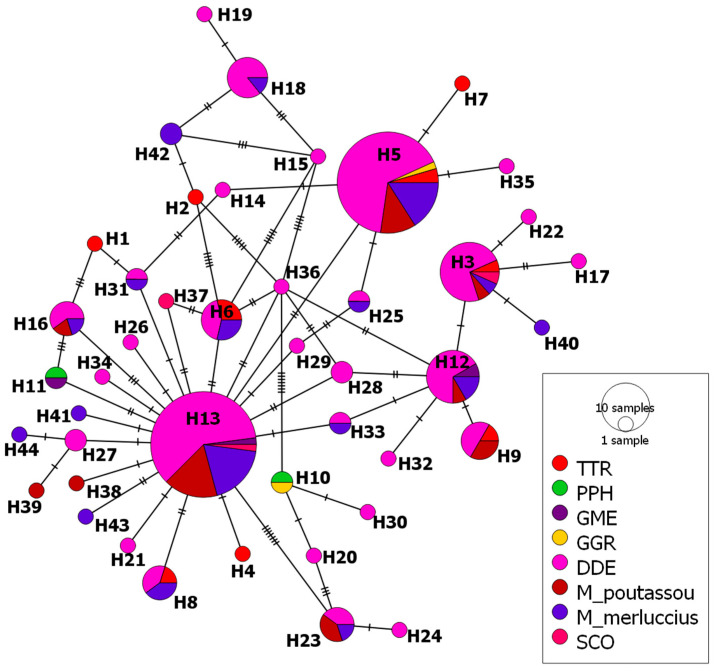
Median-joining haplotype network of *A*. *pegreffii* mtDNA *cox2* sequences obtained from cetacean and fish species from divisions VIIIc and IXa. Circles’ size represents the frequency of each haplotype. Hatch marks show the number of mutations distinguishing the haplotypes. Haplotypes shared between species are reported. Black points indicate missing haplotypes. TTR: *Tursiops truncatus*; PPH: *Phocoena phocoena*; GME: *Globicephala melas*; GGR: *Grampus griseus* and DDE: *Delphinus delphis*; and SCO: *Stenella coeruleoalba*.

Finally, haplotypes from *mtDNA cox2* sequences of A. pegreffii from cetaceans (139 sequences) and L3 larvae from M. poutassou and M. merluccius (57 sequences) were represented by a median-joining haplotype network (Figure 6). Among a total of 44 haplotypes obtained, 2 haplotypes are remarkable: H13, which was represented by 48 sequences (24.49% of total sequences), was shared between DDE, GME, SCO, *M. poutassou* and *M. merluccius*, with relative frequencies ranging between 0.246 and 0.333, while the haplotype H5, which includes 44 sequences (22.49 of total sequences), was shared by five species (DDE, GGR, TTR, *M. poutassou* and *M. merluccius*), with relative frequencies ranging between 0.182 and 0.246 for all species except TTR, for which the value was higher (0.5). Other haplotypes shared by most of the host species were H3, represented by 15 sequences (7.65% of total sequences), and H12 with 12 sequences (6.12% of total sequences). The remaining haplotypes occurred in one to three of the host species and were represented by fewer than 10 sequences. Moreover, 65.91% of the total haplotypes were exclusive to one of the eight species.

## 4. Discussion

### 4.1. Anisakis Biodiversity in Cetaceans

The Galician coast, located NW of the Iberian Atlantic coast (ICES divisions VIIIc along the north coast and IXa along the west coast), is considered an area with a high abundance and biodiversity of cetaceans and, registering a high number of stranded (mostly dead) cetaceans [44,45,46,47,48,49,50]. The most abundant resident cetacean species are *D. delphis*, *T. truncatus* and *P. phocoena* [51,52,53], although other species such as *G. griseus* and the oceanic cetaceans *G. melas* and *S. coeruleoalba* are also frequently sighted along this coast [54,55]. Moreover, this area is home to the main prey of cetaceans, such as the commercial fish *M. merluccius*, *S. pilchardus*, *T. trachurus* or *M. poutassou* [56,57,58], and around 80 cephalopod species [59].

According to the above well-established trophic links for *Anisakis* transfer [7], a recognizable established pattern of *Anisakis* biodiversity would be expected in the NW of the Iberian Atlantic coast. In fact, our analysis confirms the presence of *A. simplex*, *A. pegreffii* and their hybrids in both prey and predator species along this coast. Additionally, and for the first time, *A. berlandi* is recorded in European waters, parasitizing the long-finned pilot whales, *G. melas*; similarly, this is the first report of *A. nascettii* and *A. zhiphidarum* infecting the striped dolphin *S. coeruleoalba*.

The two members of the sibling species complex (*A. simplex* and *A. pegreffii*) were found in almost all cetacean species examined, as were hybrid genotypes. This result confirms that the NW of the Iberian Atlantic coast (IXa division) and Bay of Biscay (VIIIc) continue to be important sympatric areas for both species and their hybrids, as described in numerous previous studies since 2003, in both fish [9,15,17,18,24,25,26,60] and cetaceans [8,22]. Likewise, percentages of *A. simplex*, *A. pegreffii* and the hybrid genotype varied depending on the cetacean species. *A. pegreffii* was the predominant species in *D. delphis*, *G. griseus* and *T. truncatus*; *A. simplex* was the most abundant species in *S. coeruleoalba* and *P. phocoena* while both parasite species occurred in similar proportions in *G. melas*. Thus, *A. pegreffii* in *D. delphis* was also twice as frequent as *A. simplex* and there was a high number of hybrids. These results are different to those obtained by Cipriani et al. [8] who identified twice as many *A. simplex* as *A*. *pegreffii* and a lower number of hybrids in *D. delphis* in this area. However, the parasites identified by Cipriani et al. [8] were recovered from 3 specimens, and in our study, 30 specimens were analysed, making it likely that the present study offers a more representative view. Nevertheless, the possibility that the relative importance of the two species shifts over time should not be discounted.

The species *A. berlandi* is here reported in syntopy with *A. simplex* and *A. pegreffii* in the same individual (*G. melas*) for the first time in European waters. *Anisakis berlandi* had already been reported to be parasitizing *G. melas* in the southern hemisphere, with reports from the Chilean Pacific Ocean, New Zealand waters and the South African Atlantic coast [61,62,63]. This species was also described in the family Ziphiidae, infecting *Mesoplodon layardi* from the South African coast, *Orcinus orca* from Argentina, pinnipeds from the South Shetland Islands and several fish species from the southern hemisphere [64,65,66,67,68]. Out of its common geographic range, larvae of *A. berlandi* have also been reported in Indonesia, parasitizing the scombrid *Auxis rochei*, and in the northern hemisphere, larvae were reported in other species of cetaceans, pinnipeds and fish from the Canadian and Californian Pacific coasts and Mexico and Japan waters [7,63,66,69,70,71]. The long-finned pilot whale is a large, mainly oceanic odontocete, and is highly migratory, distributed in temperate and cold waters of the northern and southern hemispheres, and absent in tropical waters [55]. Nevertheless, based on genetic and biogeochemical markers, Monteiro et al. [72] suggested the existence of several populations in the North Atlantic, including a specific population in the northwest Iberian Atlantic coast. This ecological differentiation may be related to oceanographic phenomena and the distribution of its prey. The diet of *G. melas* is mainly composed of cephalopods (98%), with the octopod *Eledone cirrhosa* being the most abundant prey in its diet, followed by *Octopus vulgaris* and the short-finned squid belonging to the family Ommastrephidae [55]. The presence of *A. berlandi* infecting *G. melas* from the Galician coast could be explained as an accidental infection due to eating infected migratory and non-native fish species from African Atlantic waters infected with L3 larvae of *A. berlandi*. During the period from 1945 and 2022, 50 new fish species have been reported in Galician waters, based on regular monitoring since 1983 [73]. Thus, arguably, the tropicalization of the ichthyofauna of Galicia has been occurring over recent decades, associated with rising sea temperatures due to climate change [74,75]. Up to 19 species from African waters have been detected on the Galician coast in recent decades [76,77], although studies of the parasite fauna associated with these fish are scarce. Rodríguez et al. [77] described three species of nematode parasites of the genus *Hysterothylacium* and one species of the genus *Cucullanus*, none of them previously reported in European waters, in two African fish species captured in Galician waters. *Anisakis pegreffii* was also found in these fishes. Thus, the movement of fish species out of their previous geographical range could also introduce new parasites and broaden the range of host species involved, as the distribution of these parasites overlaps with those of new hosts [78,79] and/or, as in the case of *A. berlandi* reported in this study, find a common definitive host like *G. melas*.

The species *A. nascettii* and *A. zhiphidarum* are sister species according to phylogenetic analysis. They usually occur in the same hosts, with a clear preference for the family Ziphiidae, sharing their geographical range [7]. Up to now, adults of *A. ziphidarum* have been identified as parasitizing beaked whales *Mesoplodon densirostris* stranded in Galicia [80] and other species of this genus in the South Atlantic Ocean [81], the Caribbean Sea [82,83], New Zealand waters [84], Chilean waters [7] and south of the Philippines [85]. This species was also reported to infect *Ziphius cavirostris* from South Africa, Chile, the Caribbean Sea, the Gulf of Mexico and the Mediterranean Sea [7,8,83]. *Anisakis ziphidarum* has also been reported in the family Kogiidae, infecting *Kogia sima* from Philippine waters and Brazil [86,87]. *Anisakis nascettii* has been reported to infect several species of beaked whales, *Mesoplodon*, from South Africa, New Zealand and Brazil [84,88]. In this study, both species, *A. nascettii* and *A. ziphidarum*, were found, for the first time, parasitizing a member of the family Delphinidae, the striped dolphin *S. coeruleoalba*.

Data on larvae of *A. ziphidarum* and *A. nascettii* infecting teleost species in areas where their definitive hosts are present are scarce; records include several fish species but with low abundance infection values. A few larvae of *A. ziphidarum* were identified in *M. merluccius* [89], *Aphanopus carbo*, *Scomber japonicus* [90,91], *Hoplostethus cadenati* [92], *Pagellus bogaraveo* [93] and *X. gladius* [94] from eastern Atlantic coasts, from the Iberian mainland coast to Mauritania, and also from the coasts of Madeira and Azores. In Mediterranean waters, larvae of *A. ziphidarum* were also reported in *Diaphus metopoclampus* [95], *Trisopterus minutus* [96] and red scorpionfish *Scorpaena scrofa* (Scorpaenidae) [97]. *Anisakis nascettii* was identified in several fish species of the genus *Scomber*, in *Trachurus trachurus*, *M. merluccius* and *A. carbo*, across a geographical range that extends from the Iberian mainland coast and Morocco to the Azores and Canary Islands [98]. High burdens of *A. nascettii* were found in the deep-sea squid *Moroteuthopsis ingens* from New Zealand and in Tasman Sea waters [84]. This could suggest that *A. ziphidarum* and *A. nascettii* have a deep-sea life cycle. Striped dolphins are generalist predators feeding on a wide variety of pelagic and mesopelagic species, with sardine and blue whiting species being their most important prey on the western Iberian coast [99], but the low levels of parasitism of *A. ziphidarum* and *A. nascettii* in these fish suggest uncommon and accidental infections.

### 4.2. Anisakis Biodiversity in Fish

The molecular identification of *Anisakis* larvae infecting fish species in the present study showed that *A. simplex* was the predominant species, comprising up to 90% of all individuals identified, which is in accordance with previous studies [15,23]. This species was found in *Lepidorhombus* spp. from VIa, VIIb, VIIg, VIIj and in *L. budegassa* and *L. piscatorius* from the same divisions, with the exception of one hybrid, *A. simplex/A. pegreffii*, in both *Lophius* species in division VIa. Similar results were obtained for *M. merluccius* from IVa, VIa, VIIb, VIIc, VIIj and VIIh, with *A. simplex* making up between 96% and 99% of the nematodes in the digestive system and the remaining specimens being identified as hybrids. Numerous studies have shown that *A. simplex* is the only species of this genus routinely reported in waters further north than the Bay of Biscay [7,15,16,17,18], although *A. pegreffii* was also occasionally found parasitizing mackerel from the North Sea and the Norwegian Sea [15,16]. In the Atlantic waters of the Iberian Peninsula (ICES divisions VIIIc and IXa), *A. simplex*, *A. pegreffii* and the hybrid genotype had a high frequency of occurrence in *M. merluccius* and *M. poutassou*. This result provides a strong argument for the Iberian coast being an important sympatric area for both species and their hybrids [9,15,17,18,24,25,89]. The comparison of *Anisakis* species in hake and blue whiting showed similar results. Thus, *A. simplex* is the predominant species in ICES IXa for *M. poutassou* and *M. merluccius* (62.5% and 59.84%, respectively), followed by *A. pegreffii* (29.16% and 25.41%, respectively) and hybrids (8.3% and 14.75%, respectively). In the case of division VIIIc, the percentages of *Anisakis* species are also similar for both hosts, but the proportion of *A. simplex* was higher than in ICES IXa (82.86% for blue whiting and 73.23% for hake), whereas the percentage for *A. pegreffii* was lower for both fish species (13.57% for *M. poutassou* and 16.54% for *M. merluccius*). Hybrid genotypes made up 3.57% of nematodes in *M. poutassou* and 10.23% in *M. merluccius*. Pascual et al. [18] also found that prevalence values of *A. pegreffii* in hake were considerably lower in ICES VIIIc than in IXa. As might be expected, the percentages of *A. pegreffii* in hake from further north in divisions VIIIa and VIIId were even lower (4.2% and 0, respectively).

All the specimens recovered from *E. encrasicolus* from VIIIc were also identified as *A. simplex*, although the low number of individuals analysed should be considered. Other studies reported that *A. pegreffii* constituted 10.5% of total *Anisakis* identified in anchovies from VIIIc [5], and this species was also found in *E. encrasicolus* from the nearby division VIIIb, ranging between 9.7% and 47% [20,100,101].

### 4.3. Genetic Diversity and Population Structure of A. simplex

For the first time, we analysed the genetic diversity and population structure of *A. simplex* (both L3 and adults) comparing different fish and cetacean hosts. The results showed a high haplotype diversity and low nucleotide diversity for all populations evaluated. These values agree with those described in studies of the genetic diversity of *A. simplex* in other fish hosts such as *Sebastes mentella*, *M. merluccius* or *Clupea harengus* and several cetacean species from different areas from European waters [8,17,23,102]. The genetic diversity obtained for *A. simplex* is also similar to that found for this parasite in Pacific sardines (*Sardinops sagax*) from the California Current system [70].

Statistically significant highly negative values of neutrality tests, Tajima’s D and Fu’s Fs, suggest an excess of rare polymorphisms due to recent demographic expansion events and/or positive selection. These results are also consistent with those recorded in populations of *A. simplex* collected in *C. harengus* [23] and in populations of *A. simplex* from *S. mentella*, both from Northeast Atlantic fishing grounds [102].

The molecular variance analysis (AMOVA) revealed the highest proportion of intra-population genetic variation (100%) in the case of L3 larvae, as compared to values of up to 99.71% seen in adults. Similar results were obtained for *A. simplex* infecting *C. harengus* from the Northeast Atlantic [23] and for *A. simplex* adults of different cetacean species from the Iberian Atlantic coast, Scottish coast and Norwegian Sea [8]. Population pairwise Fst values showed a lack of population genetic structuring, which confirms that there is no genetic differentiation between *A. simplex* from different host species.

The 350 haplotypes obtained for *A. simplex* (L3) showed a high percentage of unique haplotypes (84%), with very low relative frequency. A single haplotype (H2) was clearly predominant, being shared by all fish species. These results indicate a clear population connection of the *A. simplex* parasitizing different fish species. The distribution of this haplotype seems even wider, as it coincides with the most frequent haplotype (H3) of *A. simplex* from *M. merluccius* from different FAO27 divisions ranging from the Iberian Peninsula to the northern North Sea [17], with the haplotype from *C. harengus* (H1) found in the Norwegian Sea, North Sea, English Channel and Baltic Sea [23] or with the haplotype (H5) for *A. simplex* usually noted all around the North Atlantic from *S. mentella* in east Greenland, the northern North Sea and the Barents Sea [102]. Likewise, this major haplotype (H2) also matches with sequences deposited in GenBank for the highly migratory fish *Mola mola* from the Mediterranean Sea [103] or *S. scombrus* from Northeast Atlantic [104]. Moreover, haplotype H2 has been obtained from common dolphins from Galicia [22], striped dolphins from the Adriatic Sea [105] and short-finned pilot whales, *Globicephala macrorhynchus*, from Biscay Bay [106]. Thus, the high frequency of this haplotype and its wide geographic and host range could indicate that it represents the most ancestral haplotype of this species [23].

All the mtDNA *cox2* sequences of *A. simplex* from fish and cetaceans from the Galician coast (NW Spain) were represented together in a median-joining haplotype network. The structure of this network is similar to that already described for *A. simplex* L3 larvae, with one haplotype (named H8), which is predominant and shared for fish species and cetaceans except for PPH, although a low number of sequences were included for that species. Haplotype H8 is the same that H3 described for L3 larvae, highlighting that is the most frequent haplotype in European waters both in different hosts and in different geographical areas. The percentage of unique haplotypes was similar to those for L3.

High genetic diversity and lack of population genetic structure have also been described for other anisakid species, such as *Contracaecum rudolphii* A parasitizing the great cormorant *Phalacrocorax carbo sinensis* from different localities from Sardinia [107], *C. rudolphii* A and B from *P. carbo sinensis* from Italy and Israel [108], *C. rudolphii* A collected from *P. aristotelis* from the Spanish Mediterranean coast [109] or *Phocanema bulbosa* parasitizing *Gadus morhua* and *Hippoglossoides platessoides* in the Barents Sea [110]. A high gene flow and a slight population genetic structure are usually reported in parasitic nematodes of fish [111] mainly due to the complex parasite life cycle with high host range and low host specificity, definitive and intermediate hosts being highly migratory and high intensity of infection in the definitive host, which come from multiple infected intermediate and paratenic hosts [8,18,68,107]. Our results also confirm previous evidence for a lack of population genetic structuring of *A. simplex* at ICES IXa and VIIIc areas [17]. However, a slight genetic sub-structuring in the *Anisakis* population from European waters was described in several studies, when different ICES divisions were compared. Thus, it has been suggested that a geographic separation of *A. simplex* was obtained both from *C. harengus* and from the minke whale *Balenoptera acutorostrata* from the Norwegian Sea [8,23]. Cipriani et al. [8] also reported a certain geographic separation in *A. simplex* collected from cetaceans of the Norwegian Sea compared with those from the Iberian Atlantic coast. Likewise, Ramilo et al. [17] described certain levels of genetic sub-structuring in the *A. simplex* population from *M. merluccius* from the northwest coast of Scotland (IVa). The arguments supporting these subpopulations are based on a geographic separation of several fish stocks which are the main food source of the cetacean species studied, as well as the presence of resident species of cetaceans in limited areas. Thus, although cetaceans are highly migratory species, they often show subpopulations with high fidelity to small areas. In Galician waters, resident populations of bottlenose dolphins, harbour porpoises and common dolphins have been described [51,52,53,58,112,113] and could be responsible for the high genetic variability and lack of genetic structure of *A. simplex* in this area.

### 4.4. Genetic Diversity and Population Structure of A. pegreffii

Our results provide evidence of *A. pegreffii* infecting *M. merluccius*, *M. poutassou* and cetacean hosts from IXa and VIIIc, with a high haplotype diversity and low nucleotide diversity for all populations. This finding is in agreement with that of Cipriani et al. [8], who recently described similar haplotype and nucleotide diversity for *A. pegreffii* recovered from different cetaceans stranded on the Iberian Atlantic coast. AMOVA and Fst tests also support the lack of population structure of *A. pegreffii* on the Galician coast. The ecology and genetic structure of *A. pegreffii* comparing different hosts from other geographical areas have already been studied. Mladineo and Poljak [114] investigated the population dynamics of *A. pegreffii* comparing three commercially important pelagic fish species (*E. encrasicolus*, *Sardina pilchardus* and *Scomber japonicus*), two demersal species (*M. merluccius* and *Merlangius merlangus*) and one top predator species (*Thunnus thynnus*) from the main Adriatic fishing grounds. These authors also evidenced the existence of genetically unstructured populations between the three groups of fish and suggested the existence of a single population that circulates between different levels of the marine food chain in the Adriatic. In this way, Blazekovic et al. [105] also indicated the absence of a genetic structure between *A. pegreffii* populations comparing different stranded toothed whales from the Adriatic Sea, based on Fst and AMOVA values, although the genetic diversity index for *T. truncatus*, *S. coeruleoalba* and *G. griseus* were much lower than those described in this study (0.75, 0.66 and 0.29, respectively). High gene flow and lack of genetic structure of population from *A. pegreffii* were also found in other hosts (*Trichiurus japonicus*) along the coast of mainland China and Taiwan [115], as well as in *Sardinops sagax* in the California Current system [70].

Our results also support the lack of population genetic structure of *A. pegreffii* in small geographical areas, even when different hosts are studied, since a high gene flow is enabled by the distribution of paratenic/intermediate hosts and the differential distribution and habitat use of dolphins, with some of them being a resident of small areas and with limitations in travelling very long distances. However, a high variability among populations from different seas worldwide was found. Significant genetic differentiation of populations of *A. pegreffii* from dolphins from the Mediterranean Sea and Iberian Atlantic waters was observed [8] and also the existence of different populations of *A. pegreffii* in the Adriatic/Mediterranean Sea, in the western Pacific Ocean and the eastern Pacific Ocean [105]. In this sense, Mattiucci et al. [116] also reported that the *A. pegreffii* population of the Adriatic and Tyrrhenian Seas was different to those of the Austral region (New Zealand and Argentine coasts).

In all mentioned studies, including ours, the high haplotype diversity is reflected in star-like phylogenetics networks, with a high number of the unique haplotypes closely related to one or two common central haplotypes, which are shared evenly between different populations of *A. pegreffii*. In this study, two haplotypes were the majority (H5 and H13) on the Galician coast but their distribution seemed to be wider since they have already been reported in several fish species such as *M. merluccius*, *M. poutassou* and *E. encrasicolus* from several Mediterranean locations [117,118,119], *M. poutassou* from the Bay of Biscay [117], *Auxis thazard* from the Canary Islands [98], *Mora moro* from New Zealand [12] or *Sympterygia bonapartii* from Argentine [120]. These haplotypes have also been previously reported in cetaceans *D. delphis* from Galicia [22], *T. truncatus* from *the* Adriatic Sea [105] and *Neophocoena asiaeorientalis* from Korea (unpublished, GenBank number MT312487).

## 5. Conclusions

This study provides novel insights into the taxonomic biodiversity of *Anisakis* spp. parasitizing cetacean species from the NW of the Iberian Atlantic coast. Thus, *A. berlandi* was detected for the first time in European waters parasitizing the long-finned pilot whales *G. melas*, and the species *A. ziphidarum* and *A. nascettii* were reported infecting the striped dolphin *S. coeruleoalba*, thus expanding their host range. *A. simplex* showed to clearly be the main identified species in fish species in FAO27, but the data reveal that in the NW of the Iberian Atlantic coast, a sympatric area for *A. simplex* and *A. pegreffii*, the latter species is on the rise, being more frequent in *D. delphis*, *G. griseus* and *T. truncatus*. Climate change and the tropicalization of the ichthyofauna on the Atlantic Spanish coast could be responsible for broadening the geographical and host range of *Anisakis* species. The genetic population structure study of *A. simplex* and *A. pegreffii* in waters off the Northeast Atlantic revealed panmictic populations for both species with high haplotype diversity, mostly maintained through stable trophic links established between the common dolphin and gadoid fishes (European hake and blue whiting).

## Figures and Tables

**Table 1:** *Anisakis* species identified infecting cetaceans stranded in Galician waters. Nh: total number of hosts; Np: total number of parasites; AS: *Anisakis simplex*; AP: *Anisakis pegreffii*; DDE: *Delphinus delphis;* SCO: *Stenella coeruleoalba*; PPH: *Phocoena phocoena*; GGR: *Grampus griseus;* GME: *Globicephala melas*; and TTR: *Tursiops truncatus*.

Cetacean Species	Nh	*A. simplex*	*A. pegreffii*	Hybrid Genotype (ASxAP)	*A. nascettii*	*A. berlandi*	*A. ziphidarum*	Np
DDE	30	84	145	43	0	0	0	272
SCO	8	50	4	6	3	0	1	64
PPH	7	20	3	3	0	0	0	26
GGR	1	1	3	2	0	0	0	6
GME	2	5	4	1	0	1	0	11
TTR	3	6	11	3	0	0	0	20
N_t_	51	166	170	58	3	1	1	399

**Table 2:** *Anisakis* species identified infecting fish species from major European fishing grounds in temperate NE Atlantic waters. Nh: total number of hosts; Np: total number of parasites; AS: *Anisakis simplex*; AP: *Anisakis pegreffii*.

	ICES Division	Nh	*A. simplex*	*A. pegreffii*	Hybrid Genotype (ASxAP)	Np
*Micromessistius poutassou*	IXa	16	15	7	2	24
	VIIIc	42	116	19	5	140
	Total	58	131	26	7	164
*Lepidorhombus* spp.	VIa	2	2	0	0	2
	VIIb	8	54	0	0	54
	VIIg	7	31	0	0	31
	VIIj	12	30	0	0	30
	Total	29	117	0	0	117
*Lophius budegassa*	VIa	3	47	0	1	48
	VIIb	6	30	0	0	30
	VIIg	6	44	0	0	44
	VIIj	4	68	0	0	68
	Total	19	189	0	1	190
*Lophius piscatorius*	VIa	4	10	0	1	11
	VIIb	6	15	0	0	15
	VIIg	1	3	0	0	3
	VIIj	4	21	0	0	21
	Total	15	49	0	1	50
*Engraulis* *encrasicolus*	VIIIc	5	5	0	0	5
*Merluccius merluccius*	Iva	5	71	0	3	74
	VIa	5	71	0	1	72
	VIIb	5	74	0	1	75
	VIIc	5	74	0	2	76
	VIIj	10	105	0	2	107
	VIIh	5	74	0	1	75
	VIIIa	5	63	3	5	71
	VIIIc	10	93	21	13	127
	VIIId	5	73	0	1	74
	IXa	10	73	31	18	122
	Total	65	771	55	47	873
Total		191	1262	81	56	1399

**Table 3:** Genetic diversity indices and neutrality tests based on mtDNA *cox2* sequences of *A. simplex* L3 from different fish species: number of sequences analysed (N), number of haplotypes (Nh), number of unique haplotypes (Nuh), nucleotide diversity (π), haplotype diversity (Hd) with their relative standard deviation (SD), average number of nucleotide differences (K), number of variable sites (S) and Tajima’s D (D) and Fu’s F (Fs) statistics with their *p*-values (D, significance level 0.05, and Fs, significance level 0.02). * significant values.

Fish Species	N	Nh	Nuh	π	Hd ± SD	K	S	Tajima’s D		Fu’s Fs	
								D	*p*	Fs	*p*
*M. merluccius*	552	230	179	0.00692	0.941 ± 0.008	3.34226	111	−2.29734	0.00000 *	−25.51202	0.00000 *
*L. budegassa*	132	77	37	0.00664	0.939 ± 0.016	3.20900	60	−2.21436	0.00000 *	−26.25170	0.00000 *
*L. piscatorius*	39	28	11	0.00621	0.946 ± 0.029	2.99730	25	−1.67671	0.03000 *	−26.28110	0.00000 *
*M. poutassou*	114	67	41	0.00708	0.952 ± 0.013	3.41981	53	−2.06895	0.00200 *	−26.16807	0.00000 *
*E. encrasicolus*	4	3	1	0.00621	0.833 ± 0.222	3.00000	6	−0.80861	0.14700	0.73089	0.57300
*Lepidorhombus* spp.	95	51	25	0.00673	0.931 ± 0.021	3.25151	45	−1.99676	0.00300 *	−26.31658	0.00000 *
Overall	936	350	-	0.00686	0.940 ± 0.006	3.31167	145	−1.84379	0.03033 *	−21.63310	0.09550

**Table 4:** Genetic diversity indices and neutrality test based on mtDNA *cox2* sequences of *A. simplex* adults from different cetacean species: number of sequences analysed (N), number of haplotypes (Nh), number of unique haplotypes (Nuh), nucleotide diversity (π), haplotype diversity (Hd) with their relative standard deviation (SD), average number of nucleotide differences (K), number of variable sites (S) and Tajima’s D (D) and Fu’s F (Fs) statistics with their *p*-values (D, significance level 0.05, and Fs, significance level 0.02). * significant values. TTR: *Tursiops truncatus*; SCO: *Stenella coeruleoalba*; PPH: *Phocoena phocoena*; GME: *Globicephala melas*; and DDE: *Delphinus delphis*.

	N	Nh	Nuh	π	Hd ± SD	K	S	Tajima’s D		Fu’s Fs	
								D	*p*	Fs	*p*
TTR	4	4	3	0.00810	1.000 ± 0.177	3.83333	7	-	-	-	-
SCO	26	21	13	0.00632	0.978 ± 0.018	2.99077	23	−1.83030	0.01800 *	−20.53535	0.00000 *
PPH	17	14	10	0.00510	0.956 ± 0.044	2.41176	13	−1.41284	0.08300	−12.30062	0.00000 *
GME	3	1	0	0.00000	-	-	0	-	-	-	-
DDE	54	33	25	0.00596	0.915 ± 0.031	2.81761	28	−1.77037	0.01100 *	−26.51293	0.00000 *
Overall	104	60	51	0.00588	0.935 ± 0.019	2.78099	46	−1.67117	0.03733	−19.78297	0.00000

**Table 5:** Genetic diversity indices and neutrality test based on mtDNA *cox2* sequences of *A. pegreffii* (L3) from different fish species: number of sequences analysed (N), number of haplotypes (Nh), number of unique haplotypes (Nuh), nucleotide diversity (π), haplotype diversity (Hd) with their relative standard deviation (SD), average number of nucleotide differences (K), number of variable sites (S) and Tajima’s D (D) and Fu’s F (Fs) statistics with their *p*-values (D, significance level 0.05, and Fs, significance level 0.02).

Fish Species	N	Nh	Nuh	π	Hd ± SD	K	S	Tajima’s D		Fu’s Fs	
								D	*p*	Fs	*p*
*M. merluccius*	38	18	12	0.00915	0.886 ± 0.038	4.41963	32	−1.46399	0.04900	−5.77758	0.02300
*M. poutassou*	22	10	4	0.00736	0.831 ± 0.063	3.55411	20	−1.30905	0.09100	−1.73765	0.19800
Overall	60	22	-	0.00845	0.860 ± 0.034	4.08023	34	−1.38652	0.07000	−3.75762	0.11050

**Table 6:** Genetic diversity indices and neutrality test based on mtDNA *cox2* sequences of *A. pegreffii* (adults) from cetacean species: number of sequences analysed (N), number of haplotypes (Nh), number of unique haplotypes (Nuh), nucleotide diversity (π), haplotype diversity (Hd) with their relative standard deviation (SD), average number of nucleotide differences (K), number of variable sites (S) and Tajima’s D (D) and Fu’s F (Fs) statistics with their *p*-values (D, significance level 0.05, and Fs, significance level 0.02). * significant values. TTR: *Tursiops truncatus*; SCO: *Stenella coeruleoalba*; PPH: *Phocoena phocoena*; GME: *Globicephala melas*; GGR: *Grampus griseus*; and DDE: *Delphinus delphis*.

	N	Nh	Nuh	π	Hd ± SD	K	S	Tajima’s D		Fu’s Fs	
								D	*p*	Fs	*p*
TTR	11	9	4	0.01039	0.964 ± 0.051	5.09091	17	−0.55029	0.31100	−2.72864	0.06600
SCO	3	3	1	0.00816	1.000 ± 0.272	4.00000	6	0.00000	0.76000	0.13353	0.28600
PPH	2	2	0	0.02449	1.000 ± 0.500	12.00000	12	0.00000	1.00000	2.48491	0.57700
GME	3	3	0	0.00544	1.000 ± 0.272	2.66667	4	0.00000	0.83000	−0.34093	0.18100
GGR	2	2	1	0.02449	1.000 ± 0.500	12.00000	12	0.00000	1.00000	2.48491	0.57500
DDE	118	34	27	0.00951	0.879 ± 0.019	4.65986	42	−1.25491	0.09600	−14.24298	0.00000 *
Overall	139	42	33	0.00979	0.896 ± 0.016	4.79575	47	−0.30087	0.66617	−2.03487	0.28083

## Data Availability

No new data were created or analyzed in this study. Data sharing is not applicable to this article.

## Supplementary Materials

The following supporting information can be downloaded at https://www.mdpi.com/article/10.3390/ani14233531/s1: Table S1: Stranding data of the 51 cetaceans from Galician coast used in this study.
